# Getting them through the door: screening optimization strategies for behavioral parent training

**DOI:** 10.3389/frcha.2025.1509235

**Published:** 2025-11-07

**Authors:** Abigail Peskin, Natalie Espinosa, W. Andrew Rothenberg, Jessica Rivera, Eileen Davis, Dainelys Garcia, Jason F. Jent

**Affiliations:** 1 University of Miami, Coral Gables, FL, United States; 2 Duke University, Durham, NC, United States

**Keywords:** parent child interaction therapy (PCIT), behavioral parent training, recruitment, referral, children

## Abstract

**Introduction:**

Clinics providing mental health treatment to children and families experience a multitude of barriers shepherding patients from their first clinic contact through to graduation from treatment, including difficulty retaining families, getting families to complete screening forms, and finding patients who are eligible for the services offered. This study describes the iterative strategies used by a mental health clinic for child behavior management training to increase families' likelihood of completing their screening forms, attending sessions, and graduating from treatment.

**Methods:**

Over the course of five years, this clinic implemented four subsequent strategies to improve intake, including introducing a structured follow-up to get patients to complete screening forms, shortening the screening forms to reduce family time burden, moving screening procedures online, and distributing a public survey link where the intake forms could be accessed without an initial phone screen.

**Results:**

Results of logistic regression analyses indicate that, although none of the screening interventions was successful for increasing a child's likelihood of attending intake or graduating from treatment, the addition of the public survey link significantly increased families' chances of completing their initial screening forms.

**Discussion:**

Findings indicate that, while other interventions are needed to improve chances of child intake attendance and graduation, it appears that the combination of screening strategies described in this study may begin to overcome barriers to families accessing treatment.

## 1 Introduction

Untreated child externalizing behavior has a high likelihood of disruptive disorders persisting into adulthood (1). Primary prevention and intervention play a key role in disrupting this trajectory, with behavioral parent training (BPT) being the most effective option for young children with externalizing concerns (2). On the journey to find effective treatment, families can be lost to attrition at many junctures: (1) between referral and contacting the agency, (2) between initial contact and completion of screening measures, (3) between initial screening and intake appointment, and (4) between intake and graduation from services. The literature that examines these stages focuses primarily on the final step, and factors that lead to attrition in treatment often include many aspects of the therapy itself, including the relationship with the therapist or active ingredients of the intervention that may be misaligned with the patient's values or goals (3). If families and children who need treatment for child disruptive behaviors are lost to attrition, particularly if this attrition occurs before any treatment has even been received, these children are at risk for a cascade of adverse outcomes, including expulsion from school, substance use and abuse, teenage pregnancy, and early death (4–6).

Successfully engaging families in treatment is a multifaceted challenge that requires both recruitment and retention efforts. Schoeppe and colleagues (7) have broken this down further into five factors that impact the success or failure of recruitment and retention, under the title of REACH, Recruit, Engage, and retAin Children in behavioral Health risk factor studies. Their literature review and interviews with experts described that there are a variety of factors that impact whether children and their families stay involved in behavioral health interventions. Factors that improve recruitment include decreasing the burden to families to access services (e.g., paperwork, etc.), work with key stakeholders in the community and project champions, and utilizing effective tools for recruitment [e.g., flyers, email, telephone; (7)]. Other factors improve engagement and retention, including providing incentives, finding preferred strategies of contact for study participants, using follow-up procedures, and providing families with feedback about study results. Others have noted that although overlap exists in effective strategies to increase both recruitment and retention, often they require a different set of skills/strategies (3). Beyond study characteristics, there are important family characteristics that impact whether they are recruited or retained in behavioral health interventions as well, including child age, caregiver socioeconomic status, family geographic area, and caregiver literacy level (7). In order to increase a child's likelihood of receiving needed behavioral health services, clinics will need to consider optimizing their procedures to improve recruitment, engagement *and* retention of families, and designing their clinic to work for the families they serve.

### 1.1 Screening completion

There is scant evidence for how or why patients are lost between initial contact and completion of screening measures. However, parallel evidence from adult behavioral health research on recruitment and retention provides some initial guidance. Factors that contributed to decreased likelihood of completing initial screening included longer form length (8), forgetting about appointments or paperwork (9), dissatisfaction with the application process (9), and limited accessibility of intake procedures (10). In contrast, increased screening completion was associated with more frequent reminders via calls or emails (9, 10) and offering the option to complete forms online rather than on paper (10, 11). These findings are consistent with REACH (7), as decreasing family burden, contacting families via their preferred methods, and increasing follow-up have been described as effective strategies for improving reach, engagement and retention in behavioral health services.

### 1.2 Intake appointments

A more robust literature exists examining the reasons why patients complete screening for services but do not attend an intake session. Indeed, approximately 25% of patients who are screened as eligible for services never make it to an intake appointment (12), and for low-income urban communities this number is even higher, ranging from 48% to 62% (3). Concerningly, patient level of distress is a factor that commonly predicts lack of intake attendance, with patients experiencing more distress being less likely to attend intake (13–15). These are often the patients with the greatest need for treatment, and in the case of child disruptive behaviors, patients whose problems are often unlikely to resolve without intervention.

The length of time that patients have to wait for services also decreases their likelihood of attending intake (15–17). However, some patients also report that by the time they complete the screening process and reach the intake stage, the issue for which they sought treatment had diminished, leading them to feel that treatment was no longer necessary (9, 18).

Caregiver characteristics also impact initial attendance for treatment. In behavioral parent training programs, caregivers of older children tend to be less likely to attend initial sessions after completing screening (19). Caregivers with more negative beliefs of their children are also less likely to attend such interventions [i.e., that their child's disruptive behavior can be attributed to causes that are internal, stable, and global; (20)], a disturbing finding considering the long-term effects of such beliefs on child behavior and the caregiver-child relationship.

Given the attrition that occurs from screening to intake appointment, some clinics have implemented innovative strategies to try to increase first appointment attendance by families. Multiple studies have demonstrated that phone contact with personalized questions about the family's concerns significantly increased likelihood of attendance at intake appointments (21, 22). Further, other research has demonstrated that reducing redundancy in steps that need to be completed prior to intake decreased the time between initial contact and intake appointment and ultimately decreased attrition (23). These findings are also supported by the REACH model (7), which describes that recruitment, engagement, and retention can also be improved by following up with families and minimizing participant burden. Here the child and family characteristics are highlighted more, as children with more intense behavioral concerns tend to be less likely to attend the first intake session.

### 1.3 Attrition during treatment

The attrition between intake session attendance and graduation from treatment has been studied extensively. This step is often referred to as engagement and retention rather than recruitment (3). Often dropout from services at this point is determined by a heterogeneous multitude of factors, including family composition [e.g., single caregiver families; (14)], changes in the presenting problem [e.g., child behavior improves; (18)], and expectations about treatment that differ from what therapy entails [e.g., expecting behavior change to occur quickly or immediately; (24)]. Due to the presence of additional barriers due to systemic imbalance in access to care (e.g., limited availability to meet for sessions, unstable housing, changes in insurance coverage), some patient demographic factors are associated with higher risk of dropout, including patient race and ethnicity [e.g., with African American and Hispanic families at a higher risk for dropout; (25, 26)], preferred language [e.g., with Spanish speaking families at increased odds of dropout; (27)], low socioeconomic status, and low caregiver educational attainment (25). Systemic barriers also become a hindrance to families' continued attendance, including access to reliable transportation, inconvenient times available for sessions, parking and childcare availability (28).

Given the high stakes of untreated externalizing behavior in childhood and the proven effectiveness of behavioral parent training [BPT; (29)], improving family retention from screening through treatment completion is essential to ensuring families receive needed care. While extensive research has explored factors influencing dropout during treatment, far less is known about why families disengage earlier in the process. Because early attrition prevents families from ever engaging with active treatment components, and disproportionately affects those most in need, it is crucial to identify and test targeted strategies to improve engagement at each transition point. A critical opportunity exists to potentially reduce disparities in access and improve long-term outcomes for children with disruptive behavior disorders.

### 1.4 The current study

The current study sought to examine four phases of strategies to increase family screening completion and its relation to attendance at intake and ultimately through graduation from treatment. Over the course of four years and four months (01/2018–04/2022), our multi-site Parent-Child Interaction Therapy (PCIT) program engaged in an iterative screening improvement process to combat the difficulties we encountered recruiting and retaining families in the community. With feedback from families who engaged in our screening and intake process and information from existing literature, we piloted and tested four phases of strategies for improving families' screening form completion and intake attendance. *Phase 1:* The follow-up process following initial contact with the clinic was standardized so that each family received phone calls to remind them to complete screening forms at consistent times after first contacting the clinic. *Phase 2:* In addition to the phase 1 strategy, the screening forms were shortened to decrease the burden of caregivers completing a long form. *Phase 3:* In addition to phases 1 and 2 strategies, the screening forms were moved online for family ease of access and completion. *Phase 4:* In addition to phases 1–3 strategies, a publicly accessible screening link on our PCIT website that briefly described the treatment was made available to families and distributed to referral partners to allow families multiple ways of contacting the clinic and beginning the screening forms.

Research question 1: Are there family level variables that impact the likelihood of PCIT patients completing screening paperwork, attending intake, or graduating from treatment?

Research question 2: Will the introduction of new screening strategies correspond to increases in the likelihood of families completing screening paperwork? Hypothesis: We predicted that with each subsequent introduction of a new screening strategy, the likelihood of families completing screening paperwork would increase significantly.

Research question 3: Will the introduction of each new screening strategy correspond to an increase in the likelihood of eligible families attending their intake session? Hypothesis: We predicted that with each subsequent introduction of a new screening strategy, the likelihood of eligible families attending intake appointments would increase significantly.

Research question 4: Will the introduction of new screening strategies correspond to an increase in the likelihood of families who attend intake subsequently graduating from treatment? Exploratory Hypothesis 3: Graduating from treatment is less likely to be impacted by procedures at screening, due to prior research indicating that engagement and retention are impacted by subtly different factors than recruitment (3). However, we explored the impact of our screening optimization strategies on graduation from treatment as well.

## 2 Method

### 2.1 Participants

Families (*N* = 2,066) referred to PCIT contacted the PCIT clinic, either via phone screen or completion of a publically-available online survey, expressing interest in the intervention. Caregivers of children ages 1–12 (*M* = 4.33, *SD* = 1.75) completed the initial screening. Although traditional treatment took place only for children ages 2–7, children were occasionally (*n* = 5) screened before their second birthdays so they could begin treatment once they turned two. Additionally, children aged 8–12 were occasionally (*n* = 76) seen if they were involved in the child welfare system, as PCIT is considered an evidence-based intervention for reducing risk of future child maltreatment (30).

Caregiver preferred language for treatment and reported race are presented in Table 1, each divided by families who reported Hispanic and Non-Hispanic ethnicity. Table 1 presents demographic information for the total sample, and divided by screening intervention utilized. The study sample consisted of 2,066 caregivers in total. The majority of participants (71.6%) identified as Hispanic, while 28.4% were Non-Hispanic.

**Table 1:** Demographics of families who completed screening forms.

Family demographics	Overall sample		Before screening interventions (01/2018–09/2018)		Phase 1: standardized follow-up (09/2018–12/2018)		Phase 2: shortened screening form (12/2018–09/2019)		Phase 3: screening moved online (09/2019–09/2020)		Phase 4: public screening link made available (09/2020–04/2022)	
Caregiver ethnicity	Hispanic	Non- Hispanic	Hispanic	Non- Hispanic	Hispanic	Non- Hispanic	Hispanic	Non- Hispanic	Hispanic	Non- Hispanic	Hispanic	Non- Hispanic
Caregiver Race	*n* = 1,104		*n* = 79		*n* = 81		*n* = 190		*n* = 292		*n* = 479	
American Indian or Alaskan	5 (0.45%)	1 (0.09%)	2 (2.53%)	0 (0.00%)	1 (1.23%)	0 (0.00%)	2 (1.05%)	1 (0.53%)	0 (0.00%)	6 (2.05%)	7 (1.46%)	4 (0.84%)
Asian	1 (0.09%)	8 (0.72%)	0 (0.00%)	1 (1.27%)	0 (0.00%)	1 (1.23%)	1 (0.53%)	2 (1.05%)	0 (0.00%)	2 (0.68%)	0 (0.00%)	2 (0.42%)
African American	11 (1.00%)	89 (8.06%)	0 (0.00%)	9 (11.39%)	2 (2.47%)	10 (12.35%)	2 (1.05%)	26 (13.68%)	0 (0.00%)	20 (6.85%)	7 (1.46%)	24 (5.01%)
White	708 (64.13%)	145 (13.13%)	48 (60.75%)	9 (11.39%)	45 (55.56%)	8 (9.88%)	110 (57.89%)	19 (10.00%)	191 (65.41%)	47 (16.10%)	314 (65.55%)	62 (12.94%)
Other	13 (1.18%)	13 (1.18%)	0 (0.00%)	2 (2.53%)	3 (3.70%)	0 (0.00%)	3 (1.58%)	1 (0.53%)	0 (0.00%)	6 (2.05%)	7 (1.46%)	4 (0.84%)
Multiracial	49 (4.44%)	38 (3.44%)	4 (5.06%)	3 (3.80%)	6 (7.41%)	2 (2.47%)	10 (5.26%)	8 (4.21%)	8 (2.74%)	6 (2.05%)	21 (4.38%)	19 (3.97%)
Haitian or ESC	0 (0.00%)	23 (2.08%)	0 (0.00%)	1 (1.27%)	0 (0.00%)	3 (3.70%)	0 (0.00%)	5 (2.63%)	0 (0.00%)	6 (2.05%)	0 (0.00%)	8 (1.67%)
Language of PCIT Delivery	*n* = 1,111		*n* = 79		*n* = 82		*n* = 191		*n* = 287		*n* = 472	
English	476 (42.84%)	305 (27.45%)	34 (43.04%)	25 (31.65%)	36 (43.90%)	24 (29.27%)	86 (45.03%)	60 (31.41%)	125 (43.55%)	85 (29.62%)	195 (41.31%)	111 (23.52%)
Spanish	304 (27.36%)	12 (1.08%)	20 (25.32%)	0 (0.00%)	22 (26.83%)	0 (0.00%)	34 (17.80%)	1 (0.52%)	71 (24.74%)	2 (6.97%)	157 (33.26%)	9 (1.91%)
English & Spanish	13 (1.17%)	1 (0.09%)	0 (0.00%)	0 (0.00%)	0 (0.00%)	0 (0.00%)	9 (4.71%)	1 (0.52%)	4 (1.39%)	0 (0.00%)	0 (0.00%)	0 (0.00%)

ESC, English-speaking Caribbean; differences in n's for different demographic categories can be attributed to caregiver demographic information being collected at differing times throughout the screening and intake process, meaning that these variables were missing for families who did not progress past screening.

Among all participants, total caregiver sample race and ethnicity are as follows: White 82.6% (71.2% Hispanic, 11.4% Non-Hispanic), followed by Multiracial at 8.4% (4.7% Hispanic, 3.7% Non-Hispanic), and Black/African American at 9.7% (1.1% Hispanic, 8.6% Non-Hispanic). Other racial categories included Other at 2.5% (1.3% Hispanic, 1.2% Non-Hispanic), American Indian or Alaskan at 1.4% (0.8% Hispanic, 0.6% Non-Hispanic), Asian at 0.9% (0.1% Hispanic, 0.8% Non-Hispanic), and Haitian or English Speaking Caribbean at 2.2% (all Non-Hispanic). Caregiver race differed significantly across some of the screening groups.

Comparatively, Miami-Dade county, the catchment area for this grant, has the following demographic breakdown per the 2020 Census (31). English is the only language spoken in 24.9% of homes, and Spanish is spoken in 66.3%. 68.7% of families identified as Hispanic, meaning there is a greater percentage of Hispanic families in our sample than the general population. 13.4% of the population identified as White non-Hispanic, meaning they were slightly underrepresented in our sample. Black/African American people represent 14% of Miami-Dade, but the Census does not differentiate between African American and Haitian or English Speaking Caribbean, so the 11.9% (9.7% African American + 2.2% Haitian/ESC) in our sample represents a slight underrepresentation of this population in our sample. Multiracial families were more difficult to ascertain, as the Census presents race and ethnicity separately. In the “non-Hispanic” population, 1.7% identified as multiracial, but then 41.9% is listed as “mixed” or “other” under race so it is unclear whether our sample (8.4%) is an over or underrepresentation of the population on this metric.

### 2.2 Procedure

#### 2.2.1 The clinic

Families in this study were referred to an academic and community-based Parent-Child Interaction Therapy [PCIT; (32)] program providing treatment to children and their families for free, funded by a county service grant. The grant-funder determines the eligibility criteria for families to receive services, and this clinic follows these criteria to ensure continued funding for free services to children and families in the community. Grant funding (and thus clinical capacity) for this clinic increased at two separate times during the four year, four month time span represented by this study. From 8/1/17 to 7/31/18 the clinic was funded to provide services to 210 families annually, from 8/1/18 to 9/30/20 for 290 families annually, and from 10/1/20 to 4/1/2022 for 340 families annually.

This program comprises six separate clinics. Four clinics are embedded within the community, within established community organizations (e.g., childcare centers, afterschool programs). These community organizations are regularly accessed by the community for many purposes, which decreases stigma for families to access mental health services in the same location. Two of the clinics are located on different campuses of the affiliated university. Locating clinics around the county also decreases the transportation burden for families to reach services, a barrier that is also addressed by providing virtual/telehealth services to families who prefer to receive services in their homes. Tech is also provided to families to increase their ability to access virtual services as needed (e.g., tablet, bluetooth headset).

PCIT is a caregiver-coaching intervention for treating disruptive behaviors in young children, with a focus on increasing the warmth and connection in the caregiver-child relationship as well as teaching caregivers to enforce healthy limits and expectations (32). All study procedures were approved by the university's Institutional Review Board. The IRB granted a waiver of informed consent for screening procedures to determine eligibility. Written informed consent was obtained from all participants at the intake session. All study procedures were carried out in accordance with the ethical standards of the IRB.

### 2.3 Phase 0: standard practice for screening, intake, then treatment

The standard screening practice (01/2018–09/2018) that was in place before additional screening strategies were added was as follows: Upon being referred, patients called or emailed their interest in receiving services. Then the screening team scheduled a phone call with them. Phone screening took place in either English or Spanish, depending on caregiver preferences. During this phone call, caregivers were asked about their child, their concerns, and their preliminary eligibility for services (e.g., residency in the county, children aged 2–7, and/or involvement in child welfare services). The grant-funder requires that children reside in the county, and the evidence-based intervention (i.e., PCIT) requires that the child be aged 2–7. If families do not qualify for services based on these first limited questions, they are not asked to complete the remaining screening paperwork, and are instead connected to services for which they would be eligible, with the goal of maximizing families' access to care and minimizing the paperwork burden for families whose children will not be eligible.

Following the phone screen, caregivers were sent a screening packet that included the screening questionnaire and an assessment about behavior (i.e., ECBI). If a child was not eligible based on the ECBI score (i.e., intensity raw score less than 131), the family was sent a BASC-3 to complete. If the BASC-3 was also not elevated on the Externalizing composite scale, families were not eligible to receive PCIT services. These eligibility criteria were determined based upon the grant received for providing this treatment for free as well as the evidence-based guidelines for PCIT, which is a treatment specialized for children with clinically elevated disruptive behavior and is overly specialized and intensive for children without clinically elevated disruptive behavior.

When all screening forms were complete, the screening team called the family to notify them of their eligibility for services. If families were eligible for services, the screening team discussed with the family their availability during the week (i.e., which day of the week and times for each day from 8 am to 7 pm) and they were placed on a waiting list for therapists to view eligible families. Families ineligible for treatment were referred out to other community agencies that also provided free services to families (i.e., funded by the same county agency or other grant-funded services).

Once the family was on the waitlist, they became visible to therapists on the team, who called families when they had available spots for treatment. Families might wait from one week to several months for treatment, depending upon their availability (e.g., families only available at 6 or 7pm typically waited longer due to few therapist openings at those times). When families were placed on the waitlist, they were given an estimate of the amount of time they would need to wait for services, and families who would need to wait longer were provided with additional referrals for other services in the community. The screening team called families about once per month to assess their ongoing interest in treatment as well as to give them an updated estimate for how long they would need to wait on the waitlist, and provided with additional referrals as requested by the family.

Internally, clinic supervisors tracked and reported to the team the progress of screening families into treatment on an approximately monthly basis. This tracking included how many families contacted the clinic and the amount that finalized their screening packets and then subsequently attended their intake sessions. When any of these metrics dropped too low (e.g., below 70%), the clinic team discussed whether a new screening procedure was needed during weekly team meetings. The clinic held a monthly recruitment meeting to review screening completion and intake attendance rates along with the existing wait lists for services. Through review of monthly data, the team jointly decided to implement new strategies when (a) screening completion rates were lower (b) there was a limited waitlist for services and/or immediate availability for specific appointment time slots.

Ideas for new screening procedures were proposed by any member of the team present in the team meetings (e.g., supervisors, clinicians, phone screening staff). Clinicians often heard from families already in treatment about ways they successfully accessed the treatment (e.g., how did they find out about PCIT, were they successful getting ahold of the clinic, did they encounter any barriers to completing screening forms, etc.) and/or challenges they faced finding treatment. Phone screening staff, who were often families' first contact with the clinic, heard from families about frustrations or difficulties they encountered throughout the screening process. Phone screening staff often were the only staff who had contact with families who did *not* complete the initial screening forms, and therefore had unique insight into the difference in experiences between those caregivers who completed the forms and those who did not. Supervisors also weighed in on these recruitment conversations, with collective insights from their various supervisees, consulting as well with literature about recruitment, retention, screening, etc. Sharing the findings from others' published studies helped the whole team brainstorm about how promising findings from others’ retention efforts that could be generalized to this community. Thus over the course of four years and four months, four new screening interventions were developed and tested. Below we explain the specific feedback from families that was pivotal in influencing each screening intervention introduced to the clinic's procedures.

### 2.4 Strategies to increase screening completion

#### 2.4.1 Phase 1: systematic follow-up procedure (09/2018–12/2018)

After the initial call to connect with families, if eligible for screening, they received a screening packet to complete (i.e., by mail, fax, email, or in-person). Before changing screening methodology, one of the recruitment concerns identified by the clinical team was that families often did not complete and/or return their screening packets. At some point after not receiving the packets, the screening team followed up with the family, but this process was not structured or standardized. Phase 1 included all of Phase 0 screening procedures but also the addition of systematic follow-up. New methodology established in September 2018 dictated that the screening team would call the family two weeks after sending the screening packet to determine whether they required assistance completing the forms. All communication (i.e., phone calls, emails, etc.) were completed by bilingual (i.e., English and Spanish) screening team members, and were conducted in the family's preferred language. The REACH framework indicates that screening teams thus trained in communication with the clinic's families, as well as providing communication in a family's preferred modality, are factors that can increase recruitment and retention (7).

#### 2.4.2 Phase 2: shortening the screening questionnaire (12/2018–09/2019)

Next, families expressed frustration with the amount of forms and questions they had to complete before being determined eligible and placed on the waitlist or referred out to other services. During the screening team's follow up calls, families' feedback was often that they had not yet completed their forms due to the length. The first strategy attempted to address this barrier to screening completion was to give families the option of completing the forms over the phone with a member of the screening team, as some families took a long time to complete online due to low literacy levels. This strategy aligns with the REACH principle of communicating with families in a way that works for them (7). However, few families chose this option, as it often took more time than completing it independently (i.e., the screener had to read each question and discuss the answer).

This screening questionnaire was also utilized inefficiently between specialists (e.g., pediatricians, psychologists, speech-language pathologists) in the larger academic medical center where this clinic was located. Every individual clinic within the center had its own questionnaire, so if a family was referred from the developmental and behavioral pediatrician to the psychologist two doors down (for example), they would be required to complete a new intake questionnaire, thus decreasing their likelihood of following through with a referral to another professional due to the time burden completing forms [i.e., another REACH recommendation for increasing recruitment and retention, (7)]. Therefore, in December 2018, in collaboration with the other specialists in the center, the PCIT clinic created a common screening form that decreased in length (i.e., from 12 pages to 8) and time to complete (i.e., from approximately 40 to 30 min). This procedure was the newest screening addition for about nine months until a new change was made.

#### 2.4.3 Phase 3: moving screening online (09/2019–09/2020)

Prior to 2019, families received their screening questionnaires by email, fax, mail, or in-person, and had the option to return them via the same delivery method. In September 2019, the clinic began to utilize Research Electronic Data Capture [REDCap; (33)], a HIPAA compliant electronic data collection software, to enter and analyze data. Study data were collected and managed using REDCap electronic data capture tools hosted at the University of Miami (33, 34). Soon after integrating REDCap into the clinic, the screening team began to utilize it to send screening questionnaires to families. Thus, families received a link that opened in their web browser on computers or other devices and they securely completed the information there which would then be automatically sent back to the screening team (via REDCap). This eliminated the need for families to download or print forms that were emailed to them, or to mail them, fax them, or bring them back. For families who were not able to complete assessments virtually, they had the option to request that they either complete them on paper, or verbally over the phone with the screening staff. This step was another attempt to minimize participant burden for completing screening forms, consistent with REACH (7).

#### 2.4.4 Phase 4: online link for screening (09/2020–04/2022)

In September 2020, the phone screen remained a family's gateway into screening and thus treatment. However, some families found phone screenings difficult to schedule inside of clinic hours (e.g., treatment sessions are sometimes offered at 8 am or 6 pm, but phone screenings typically took place between 9 am and 5 pm). If a phone screening could not be completed, there was no other way to access services at this particular clinic. Once families completed the phone screen, however, screening questionnaires could be completed entirely online at whatever time they chose. Therefore, another way to streamline the screening process was to give families access to the screening questionnaires to complete prior to completing the phone screen. This alternative strategy for clinic initial contact is consistent with the REACH strategies of using communication strategies that work for the family as well as consideration of family-level factors (e.g., caregiver work schedule) that could impact screening paperwork completion.

Logic branching was embedded in the online screening such that if families indicated specific parameters that automatically ruled them out (e.g., child age, geographic location, presenting concerns), they were not asked to complete the remainder of the form, with the goal of minimizing participant burden [i.e., REACH strategies, (7)]. This format was combined with a QR code that could be scanned for easier access to the public link. This code was then added to flyers, so that the public screening link was available in a variety of settings, including caregiver workshops, community events, etc. Including the link in resources shared by other organizations fits with the REACH strategy of creating community partnerships with key stakeholders to improve recruitment and retention as well (7). Once their screening information was completed online, the same process occurred for contacting families about eligibility; the screening team called them (using contact information they provided during the online screening process) to inform them of their eligibility (or non-eligibility) and either placed them on the waitlist for treatment or referred them out to community partners who also provided free, grant-funded services. Specific, tailored recommendations to community partners were provided which matched the caregiver's expressed concerns as well as scores on eligibility assessments completed during the screening.

### 2.5 Measures

**Screening Form.** In addition to assessments about child behavior, during the screening process caregivers completed forms providing information about the family, including information about birth (e.g., complications), previous assessments and treatments, and family medical history. Families also provided availability for treatment sessions, limited demographic information (e.g., language, child age), and general behavioral areas of concern.

Phase 0–1: The screening questionnaire in phases 0 and 1 consisted of a 12-page document asking a variety of questions about child behavior, developmental history, assessments, and screening for symptoms of autism. Phase 2–4: The screening questionnaire was shortened to 8 pages, and included more yes/no questions for caregivers to complete instead of fill-in-the-blank options which required more time.

**Eyberg Child Behavior Inventory** (ECBI; 35). The ECBI is used in order to assess eligibility for treatment using caregiver report of conduct and behavioral problems in children and adolescents ages 2–16. The assessment measures the number of difficult behavior problems and the frequency with which they occur. Studies have indicated good reliability and validity for the ECBI in racially/ethnically diverse populations [*α* = 0.94, test–retest = 0.75; (36)], including stability over time and sensitivity to treatment-related changes. This caregiver report instrument takes five minutes to complete and five minutes to score. For the purposes of this study, the raw Intensity score was used to assess eligibility for treatment [i.e., a sum of the 36 items asking about the frequency of problem behaviors on a likert scale of 1 (never) to 7 (always)], with children scoring >= 131 determined eligible.

**Behavior Assessment System for Children**, **Third Edition Parent Rating Scale** [BASC-3 PRS; (37)]. The BASC-3 (i.e., subscales for Hyperactivity, Aggression, and Conduct Problems, and Composite Externalizing Problems scale) was used to assess eligibility for treatment using caregiver report of children's externalizing, internalizing, and adaptive behaviors in children and adolescents ages 2–21. The BASC-3 is a norm-referenced, standardized behavioral assessment system designed to facilitate the differential diagnosis and classification of a variety of emotional and behavioral disorders in children. Research has demonstrated good reliability and validity for the measure. For the purposes of this study, the *T* scores for Externalizing subscales (i.e., Hyperactivity, Aggression, and Conduct Problems) and composite Externalizing Problems score were used to assess eligibility for treatment if the child did not meet ECBI eligibility. Children were determined eligible if they scored a *T* score of 60 or higher on one or more of these scales.

### 2.6 Data analysis

Descriptively, we examined the time period prior to and following each of the four screening optimization strategies, to determine the average percentage of complete and incomplete screenings per month. Quantities and percentages are presented in Table 3 of families who: (a) completed phone screens, (b) completed screening forms, (c) attended intake and (d) graduated from treatment for each time point of screening intervention. Across time points from phone screen to graduation, the denominator used to calculate the percentage was consistently the number of families from the previous cell. So for instance, in the first row of Table 3, to calculate the percentage of families who completed screening forms, we divided 107 families who completed forms by 168 total phone screens. Then, to calculate the percentage of families who attended intake, we divided 82 families who attended intake by the 107 families who completed forms.

**Research question 1.** REACH suggests that recruitment, engagement and retention can often be significantly impacted by family demographic factors (7). To ensure that significant findings could be attributed to the screening interventions themselves (and not other demographic characteristics), first chi-square analyses for dummy coded variables (see Table 2) were completed between the predictors (i.e., Phases 0–4 of screening optimization) and outcomes (i.e., screening packet completed, intake attended, graduation) and available demographic data.

**Table 2:** Pearson chi squared analyses comparing predictors and outcomes with patient demographics.

Family demographics	Phase 0	Phase 1	Phase 2	Phase 3	Phase 4	Completed screening forms	Attended intake	Completed treatment
1. Caregiver ethnicity	.380	.018	1.663	.541	4.111*	.168	4.596*	1.104
2. Race: White	1.236	6.894**	11.341**	7.785**	4.380*	.401	2.920	7.707**
3. Race: Black	.495	3.517	8.985**	1.998	5.843*	4.519	6.400**	13.828**
4. Race: Asian	.328	.299	2.245	.007	2.799	.284	.022	2.207
5. Race: Haitian/ESC	.246	1.249	.432	.011	.875	2.213	1.287	1.401
6. Race: Multiracial	.107	.468	.779	4.827*	.557	3.287	.182	.017
7. Race: Other	.007	.648	.080	.142	.006	.016	.024	.221
8. Race: Am. Indian	6.090	.745	4.431	2.152	4.288*	.212	1.010	1.154
9. Tx Lang.: English	2.067	.158	.013	1.621	3.683*	1.646	1.793	8.872**

Only correlations of sociodemographic characteristics with predictor and outcome variables are shown given the focus of the study.

Caregiver ethnicity: 0 = Non-Hispanic, 1 = Hispanic.

Caregiver race is represented by seven dummy coded variables in which the variable name (Race: White, Race: Black, etc.) is coded 1, and all other races are coded 0.

Treatment Language: English is coded 1 = English only, 0 = Spanish and Bilingual (i.e., family requested a therapist who was bilingual in Spanish and English).

Phase 0 = prior to screening optimization strategies being introduced; Phase 1 = streamlining follow-up strategies; Phase 2 = Shortened intake form; Phase 3 = Moving screening online; Phase 4 = Public screening link available.

* *p* < .05.

** *p* < .01.

**Table 3:** Number and percentage of families who completed phone screens, screening forms, intake appointments and treatment graduation at each time point.

Phases	Initial contact with clinic N/month (N)*	Screening forms completed n/month *n* (%)	Eligible for treatment n/month *n* (%)	Attended intake appointment n/month *n* (%)	Graduated treatment n/month *n* (%)
Phase 0: Original Approach (01/2018–09/2018)	21.00/month 168	13.38/month 107 (63.69%)	12.88/month 103 (96.26%)	10.25/month 82 (76.64%)	6.75/month 54 (65.85%)
Phase 1: Systematic Follow-Up (09/2018–12/2018)	60.33/month 181	35.67/month 107 (59.11%)	29.67/month 89 (83.18%)	24.33/month 73 (68.22%)	14.33/month 43 (58.90%)
Phase 2: Shortened Intake Form (12/2018–09/2019)	45.67/month 411	30.78/month 277 (67.40%)	25.78/month 232 (83.75%)	19.33/month 174 (62.82%)	12.11/month 109 (62.64%)
Phase 3: Screening Moved Online (09/2019–09/2020)	45.25/month 543	31.50/month 378 (69.61%)	24.83/month 298 (78.84%)	20.25/month 243 (64.29%)	14.42/month 173 (71.19%)
Phase 4: Public Survey Link (09/2020–04/2022)	66.83/month 1203	49.50/month 891 (74.06%)	34.50/month 621 (70.00%)	26.00/month 468 (52.53%)	16.33/month 294 (62.82%)

N = total families per phase who made initial contact with the clinic. Phases 0–3, initial contact required an initial phone call to the clinic to express interest in treatment. Starting in Phase 4, initial contact could mean either a phone call or completing the online public screening link; n = the number of caregivers who completed the stage (screening forms completed, eligible for treatment, intake attended, and graduated treatment; given that phases differed in length of time, average completions per month are provided for comparable metrics; % refers to the percentage of caregivers who completed that step in the treatment process relative to the previous step.

Screening forms completed % = screening forms completed n/phone screens completed *N*.

Eligible for treatment % = eligible for treatment n/screening forms completed *n*.

Attended intake appointment % = attended intake appointment n/ eligible for treatment *n*.

Graduated treatment % = graduated treatment n/attended intake appointment *n*.

Number per month is calculated by taking the *n* of the cell and dividing it by the number of months for each phase (Phase 0 = 8 months; Phase 1 = 3 months; Phase 2 = 9 months; Phase 3 = 12 months; Phase 4 = 18 months).

**Research questions 2–4.** Further, logistic regression analyses were conducted to determine whether any of the specific methods for screening optimization (Phases 1–4) uniquely increased the likelihood of families (a) completing screening forms, (b) attending intake sessions, and (c) graduating from treatment (38). Prior to completing these regression analyses, researchers ensured that assumptions were met for a logistic regression model. First, all dependent variables were determined to be coded 0/1. Second, no predictor could be shown to perfectly predict the outcome (i.e., perfect separation). Third, predictors that were collinear with predictors (i.e., phases) were detected and excluded from regressions (see above for more detail). Sufficient sample size per predictor (i.e., greater than 10 events per parameter (39) was ensured before predictors were included in the regression model (38).

Given the concern about cohort effects, several other variables were considered for inclusion in the subsequent regression analyses, particularly variables that may have differed between Phases 0–4. These included whether families received virtual or in-person services, whether treatment took place during the COVID-19 pandemic or prior, and whether screening/treatment took place prior to or following two separate iterations of funding increase (8/1/2017–7/31/2018 funding for 210 families annually; 8/1/2018–9/30/2020 funding for 290 families annually; 10/1/2020 and later funding for 340 families annually). However, upon examining collinearity between these cohort variables and the Phases, multicollinearity was determined to be too high and so these variables were excluded. Limitations to such exclusion are explored in the limitations section below.

In each of the three logistic regressions, each screening strategy (i.e., predictor) was dummy coded. For example, in Table 4, the public screening link was coded 0 if a participant did not receive the suite of strategies that included the public screening links and 1 if they did do so. Importantly, because this same dummy coding procedure was used for all 4 strategies, and all 4 dummy-coded variables were included in the model at once that means that the logistic regression odds ratios can be interpreted as the increase or decrease in odds of completing a particular part of the study compared to being in the original approach group that received no such strategies. Also, it is important to keep in mind that these strategies were implemented sequentially and cumulatively. So, for instance, the “public screening” link variable does not mean that the group of participants coded 1 in this variable only received the public screening link. It means that those participants received the public screening link *and all other engagement strategies that preceded it in implementation* (i.e., moving the screening online, shortened intake, and follow-up call). This is true for all variables.

**Table 4:** Results of regression analyses*.*

Predicting completion of screening packet					
Screening intervention strategy utilized	*n*	B	S.E.	P	OR
Phase 1: Follow-up call	107	-.202	.220	.358	.817
Phase 2: Shortened Intake	277	.155	.192	.419	1.168
Phase 3: Moving screening online	378	.258	.185	.164	1.294
Phase 4: Public screening link	891	.478	.173	.006	1.613
Predicting caregiver attendance at intake session					
Screening intervention strategy utilized	*n*	B	S.E.	P	OR
Phase 1: Follow-up call	73	−1.194	.689	.083	.303
Phase 2: Shortened Intake	174	−.748	.653	.252	.473
Phase 3: Moving screening online	243	−.653	.639	.307	.521
Phase 4: Public screening link	468	−.423	.625	.499	.655
Caregiver ethnicity		−.563	.309	.068	.569
Predicting graduation from treatment					
Screening intervention strategy utilized	*n*	B	S.E.	P	OR
Phase 1: Follow-up call	43	−.365	.370	.324	.694
Phase 2: Shortened Intake	109	−.214	.312	.492	.807
Phase 3: Moving screening online	173	.042	.304	.891	1.043
Phase 4: Public screening link	294	.056	.286	.845	1.057
Caregiver race: White		.316	.214	.140	1.372
Caregiver race: African American		−.926	.285	.001	.396
Language of treatment		.453	.166	.006	1.573

Caregiver race: White coded 0/1 so White = 1. Caregiver race: Black coded 0/1 so African American = 1.

Caregiver ethnicity: 1 = Hispanic, 0 = non-Hispanic.

Language of treatment: 1 = English, 0 = Spanish and Bilingual (i.e., Spanish & English).

## 3 Results

### 3.1 Summary of initial descriptive findings

Descriptive findings for number, percentage, and average per month of participants at each phase who contacted the clinic initially, completed screening paperwork, were eligible for PCIT, attended intake, and graduated from treatment are presented in Table 3. Taken together, these descriptive findings preliminarily indicate that the cumulative addition of new screening interventions might have increased the likelihood of participant completion of screening packets, as completion percentages increased from 63.69% before these interventions were implemented to 74.06% after the final intervention (the online link for the screening survey) was implemented. However, it does not appear that these interventions improved percentages of completion of intake appointments (as 76.64% of those who completed the screening packets attended intake appointments before the interventions were implemented, and 52.53% did so after) or treatment completion (as 65.85% of those attended intake sessions completed treatment before the interventions were implemented, and 62.82% did so after). However, these initial descriptive findings needed to be empirically evaluated to see if these differences in completion percentages were meaningful after controlling for demographic characteristics of the sample, and whether they were statistically significant. That empirical evaluation is described below.

#### 3.1.1 Research question 1

See Table 2 for chi square analyses comparing demographic variables, predictors, and outcomes. Screening packet completion did not differ significantly by caregiver race or ethnicity, or language of treatment. However, several significant findings were revealed regarding demographic correlates of child attendance at intake and family graduation.

Child attendance at intake varied significantly by child ethnicity [*x*^2^(1, 1,111) = 4.596, *p* = .032; Hispanic families were less likely to attend intake]. Child ethnicity was therefore included in further analyses predicting intake attendance. Family graduation from treatment varied significantly by treatment language [*x*^2^(1, 2,507) = 9.034, *p* = .011; English-speaking caregivers more likely to graduate] and race (White families more likely *x*^2^[1, 1,076] = 7.707, *p* = .007; and African American families less likely to graduate, *x*^2^[1, 1,076] = 13.828, *p* < .001). Therefore, child race (only White and African American dummy coded variables) and language of treatment were subsequently included in analyses predicting graduation.

Following preliminary analyses of demographic covariates, separate logistic regressions were conducted to determine which independent variables contributed to (1) screening forms being completed, (2) intake appointment attended, and (3) treatment completion (38).

#### 3.1.2 Research questions 2–4

The binary logistic regression examining the effects of the screening optimization strategies on the likelihood of screening form completion produced an overall significant model, *x*^2^(df = 4) = 23.542, *p* < .001. The model accounted for 1.3% of the variance (Nagelkerke *R*^2^). Table 4 presents the findings for individual predictors. The binary logistic regression model examining the effects of the four phases of screening strategies on the likelihood of attending intake produced an overall nonsignificant model, *x*^2^(df = 7) = 9.603, *p* = .212. The model accounted for 2.5% of the variance (Nagelkerke *R*^2^). The binary logistic regression model examining the effects of the four phases of screening strategies on the likelihood of graduating from treatment produced an overall significant model, *x*^2^(df = 7) = 37.466, *p* < .001. The model accounted for 5.7% of the variance (Nagelkerke *R*^2^). See Table 4 for statistics of individual predictors.

The only significant finding between screening interventions and treatment time points indicates that families who were screened during the time when the public survey link was introduced (and therefore received the public screening link, had their screening online, had a shortened intake form, and received systematic follow-up) were significantly more likely to complete their initial screening forms compared to families who received the initial approach. For families who completed their screening forms, none of the specific interventions to optimize screening significantly increased the likelihood of attending the intake appointment. Additionally, for the families who attended the intake appointment, none of the specific interventions significantly increased the likelihood of graduating from treatment. However, as demonstrated in Table 3, though the screening interventions did not make it more likely for families to attend intake or graduate from treatment, it appears screening procedures did vastly expand the *number* of families who attended intake and graduated treatment. Because over 3 times as many families were being screened per month during the time when the Public Survey Link was available (compared to the original approach), almost 3 times as many families attended intake appointments and graduated treatment (compared to the original approach). However, it is important to note that this increase coincided with a 1.68-fold increase in funding during the same period, which allowed the clinic to serve more families annually. Therefore, the improvements in screening and intake attendance are likely the result of a combination of factors, including both the implementation of the Public Survey Link and the availability of additional resources.

## 4 Discussion

Mental health clinics spend an inordinate amount of time screening families for eligibility for treatment, getting them to intake (e.g., by calling to follow up with them, trying to make the waitlist a reasonable length, etc.), and then working to get them to graduation. However, despite all of this time and effort, many families are lost to attrition, sometimes before they have even begun (12). Therefore, knowledge about why and how families progress successfully from screening to graduation is needed. Often factors that influence this progression are outside the control of the clinic [e.g., family previous experiences with mental health; (40)]. This study endeavored to discover if specific clinic screening/intake procedures could measurably impact families' progress through the screening and treatment process.

The only predicted finding that emerged as significant was the addition of the public survey link to the screening process, which significantly increased the odds that families completed their screening forms. Notably, the public survey link was the last in a series of interventions to improve the screening process for families. The previous three interventions did not significantly increase the number of families screened, but the addition of the public survey link represents a cumulative effect of several layered quality improvements partially dependent on the effects of the others. This need for multiple strategies to increase recruitment, engagement, and retention is supported by prior research (3, 7).

The public survey link in particular might serve as an especially effective way to ensure families complete screening forms because it serves as a “one-stop shop” that allows immediate action at a time a family is most motivated to seek treatment (i.e., right when they see a flyer or hear a referral). The public survey link capitalizes on families' immediate enthusiasm for treatment when they see it advertised by allowing them to submit screening materials right away. It does not require families to sustain interest in a treatment and find the motivation to call or complete an online form later. Therefore this strategy aligns with the conceptual motivations behind other engagement strategies that decreased time between initial contact and first client action [e.g., (22, 23)]. By immediately capitalizing on families' initial motivation to seek treatment, public survey links drastically reduce time between initial contact and first client action. An additional benefit of the public survey link is that the survey itself could be embedded into the team's existing REDCap, and a QR code shared on flyers and with other stakeholders/providers in the community meant that hosting the public survey link did not incur any further costs for this clinic.

Of note, none of the four interventions tested significantly affected the odds of families attending an intake session or graduating from treatment. Consistent with prior research on increasing retention in behavioral health treatments, clinic logistical/administrative interventions like those described in this study are often more effective for initial contact with families. However, individual progression through treatment often varies more based on family and therapy-specific factors that were not systematically manipulated by these interventions [e.g., rapport with therapist, therapist's cultural humility; (3, 40)]. The REACH model discusses that intake attendance *may* be positively impacted by strategies such as minimizing participant burden, improving follow-up, and tailoring communication strategies to meet the families' needs, all of which were utilized in the screening strategies in this study. However, the lack of increased likelihood of intake attendance indicates that other factors are more powerful indicators of whether families will attend intake, like features of the child or family itself.

Consistent with prior literature (41), several family sociodemographic characteristics differed between families who attended intake and those who did not, and those who graduated from treatment vs. those who terminated prematurely. For families' likelihood of graduating treatment, these demographic variables were some of the strongest predictors in the overall model. Families with African American caregivers and those who received treatment in Spanish were significantly less likely to graduate compared to the other families. Findings for African American caregivers reflect those of some prior studies of BPT interventions, indicating that the skills taught in PCIT may not resonate as well with the parenting values of African American caregivers (42, 43). Additionally, although PCIT has been demonstrated to be acceptable and effective in a Spanish-speaking sample (44), Spanish speaking therapists may provide PCIT in subtly different ways which impact attrition, like their strategies for coaching caregivers (45). Finding that caregivers who receive treatment in Spanish are significantly less likely to graduate indicates a pressing need for more research to understand the mechanisms underlying this relationship.

Given the amount of energy that clinics place on recruitment and screening potential families, these findings suggest that incorporating an electronic public survey link could increase the clinic's efficiency of completing the screening process. Specifically, it would provide families with an opportunity to complete forms quickly, thus allowing the clinic to determine program eligibility sooner. Having the ability to determine eligibility swiftly would also allow programs to have a more accurate representation of their waitlist and approximation for service wait time. In turn, this would allow the clinics to resolve the status of families on the waitlist as well as improve clinic workflow, efficiency, and timeliness. In addition, having families complete electronic public surveys would provide clinics with the opportunity to have a more complete client medical record (EMR) with improved accuracy.

### 4.1 Screening interventions as a way to extend the reach of interventions?

Though we did not hypothesize this trend *a priori*, it was notable to us as we examined Table 3 that the addition of screening interventions dramatically increased not only the number of phone screens completed per month compared to our original approach, but also increased the number of intake appointments and families that graduated treatment per month between two and three times compared to our original screening approach. Therefore, it appears that our screening strategies also hold potential to make it easier for more families to enter the service pipeline. So, even if these screening strategies do not increase the percentage of families completing intake or graduation, they still increase the sheer number of families who are being served beyond what would be expected relative to funding increases, which ultimately reduces mental health burden in communities (23, 46).

Notably, many of the increased families who completed screening paperwork were determined to be ineligible for PCIT and thus referred out. To increase the family's likelihood of attending mental health services, other clinics could improve this model by offering a wider range of available services, as it would decrease the family burden to receive whatever services they needed under one umbrella rather than having to start the screening process over again at a new clinic once they were not eligible (7).

### 5 Limitations and future directions

Several methodological limitations warrant consideration. First, this study is meant to analyze the attrition that takes place at unique points in the screening and treatment process, but as a result neglects what happens during the course of treatment itself. However, the aim of this study was also to see whether logistical screening-related factors would impact attrition. Often dropout that occurs during the course of treatment is found to be more related to factors related to the treatment itself and its fit with the family's view of the cause of behavior (47) and external barriers to weekly attendance and practice (48, 49). There is little likelihood that the screening interventions described here would affect treatment drop, based on previous literature described above as well as this study's finding that treatment completion itself was not significantly impacted by any of the described interventions. However, future studies may want to examine more session by session effects to determine at what point clinical and therapy-specific factors overrule those related to the process and ease of screening completion.

The only significant screening intervention strategy that emerged was the use of the public survey link to increase completion of the screening forms. However, the use of the public survey link differs from the other three implementation strategies in one crucial way. That is, for the follow-up standardization, shortening the intake form, and moving screenings online, the intervention was applied to all new participants right when they were introduced. The public survey link was only utilized by those who chose to begin their screening forms this way; others could elect to call the clinic instead. Therefore, although the introduction or availability of the public screening link significantly increased screening forms completion, it is not clear which of the families in this group actually used the public screening link and which called the clinic to complete the phone screen.

This study was an examination of iterative improvements that a clinic made over five years to decrease the burden of intake processes for the families trying to reach services. Therefore, the adjustments made to screening procedures were not administered in a systematic manner conducive to clear-cut research conclusions. Many of the adjustments were made as a result of feedback from families, and could therefore not have been planned from the beginning of when data were collected. One of the largest downsides to this study design is that after the first screening streamlining procedure was introduced (i.e., the systematic follow-up calls), none of the screening interventions were tested on their own. In other words, they were introduced cumulatively, and everyone who got number 3 (the online screening) also received number 2 (the shortened intake form), etc. Future research would benefit from testing them separately and may be able to discern whether any differences were due to the screening intervention itself, the passage of time, or other history-confounded factors.

Another substantial limitation of this study regards the potential for cohort effects to impact results in substantial ways (50). That is, each of the screening optimization strategies was introduced at different points in time, and not compared to one another at the same time. Unfortunately this could not be avoided in this clinic given the strategies were changed for clinical/family reasons rather than to answer research questions. This means that at the timing of each new screening strategy, the clinic may have also provided different services regarding the therapists available, screening team encountered, time on waitlist, etc. Families may have also experienced differing stressors of their own from larger worldwide (e.g., COVID) or time of year (e.g., summer vs. school year) variables which differed between the phases of screening optimization. Often it was not possible to include these variables in analyses, as the “events per variable” were smaller than 10, which can negatively impact the accuracy of the results (39), or the covariates were too highly collinear with the existing predictors (i.e., phases of screening optimization) to be added to the logistic regression without violating the assumptions of the regression model. For example, funding increased once during the time span from the first to the last screening optimization strategy introduced, a clinical difference which may have changed how quickly families started intake (i.e., less time on the waitlist), or how much the screening team was able to support them through the screening process. The presence of the COVID-19 pandemic and subsequent precipitous increase in virtual services may have changed the families who attended services, but these variables occurred in the same time as the phase variables so they had to be excluded.

Importantly, the separate “cohorts” represented (i.e., the separate samples in each of the four phases) also demanded distinct screening methods that gave rise to the new screening optimization strategies. As the new strategies introduced were informed by clinic flow (e.g., number on the waitlist) and family expressed needs (e.g., shorter screening forms), each cohort expressed a new need for screening to be different and thus the cohort is inextricably linked with the new screening strategy introduced during its tenure. That said, these cohort effects should be taken into consideration when attempting to replicate any findings or make larger generalizations to practice.

## 6 Conclusion

Taken together, these results represent an important addition to our understanding of how mental health clinics can get families from their initial contact to completing service eligibility forms. Although a less-studied area in the pipeline of access to treatment, this is nonetheless a point at which families are lost to attrition, thus increasing the children and families who do not access needed care. Clinics who utilize the successful strategy (i.e., public survey link combined with the prior strategies) described here may further decrease the number of children who go untreated, and hopefully thus decrease the risk of those children developing further negative outcomes later in life.

## Data Availability

The raw data supporting the conclusions of this article will be made available by the authors upon request.

## References

[1] Reef J Diamantopoulou S van Meurs I Verhulst FC van der Ende J. Developmental trajectories of child to adolescent externalizing behavior and adult DSM-IV disorder: results of a 24-year longitudinal study. Soc Psychiatry Psychiatr Epidemiol. (2011) 46:1233–41. 10.1007/s00127-010-0297-9 20936464 PMC3214259

[2] Forehand R Kotchick BA. Cultural diversity: a wake-up call for parent training –republished article. Behav Ther. (2016) 47(6):981–92. 10.1016/j.beth.2016.11.010 27993345

[3] McKay MM Bannon WM Jr. Engaging families in child mental health services. Child Adolesc Psychiatr Clin N Am. (2004) 13:905–21. 10.1016/j.chc.2004.04.001 15380788

[4] Sayal K Washbrook E Propper C. Childhood behavior problems and academic outcomes in adolescence: longitudinal population-based study. J Am Acad Child Adolesc Psychiatry. (2015) 54(5):360–8. 10.1016/j.jaac.2015.02.007 25901772

[5] Copeland WE Wolke D Shanahan L Costello EJ. Adult functional outcomes of common childhood psychiatric problems: a prospective, longitudinal study. JAMA Psychiatry. (2015) 72(9):892–9. 10.1001/jamapsychiatry.2015.0730 26176785 PMC4706225

[6] Hicks BM Iacono WG McGue M. Identifying childhood characteristics that underlie premorbid risk for substance use disorders: socialization and boldness. Dev Psychopathol. (2014) 26(1):141–57. 10.1017/S0954579413000862 24280373 PMC3946375

[7] Schoeppe S Oliver M Badland HM Burke M Duncan MJ. Recruitment and retention of children in behavioral health risk factor studies: REACH strategies. Int J Behav Med. (2014) 21:794–803. 10.1007/s12529-013-9347-5 24198037

[8] Hoerger M. Participant dropout as a function of survey length in internet-mediated university studies: implications for study design and voluntary participation in psychological research. Cyberpsychol Behav Soc Netw. (2010) 13(6):697–700. 10.1089=cyber.2009.0445 21142995 10.1089/cyber.2009.0445 PMC4367493

[9] Benway CB Hamrin V McMahon TJ. Initial appointment nonattendance in child and family mental health clinics. Am J Orthopsychiatry. (2003) 73(4):419–28. 10.1037/0002-9432.73.4.419 14609404

[10] Kovach JV Flores MN. Streamlining admissions to outpatient substance use treatment using lean methods. J Subst Use. (2021) 26(3):306–12. 10.1080/14659891.2020.1821809

[11] Sook Chun R Edison DL Cozza SJ Cox A Patel SP Parker W Implementation and Evaluation of a Web-Based Automated Mental Health Intake System. Fort Detrick, MD: U.S: Army Medical Research and Materiel Command (2002).

[12] Chacko A Jensen SA Lowry LS Cornwell M Chimklis A Chan E Engagement in behavioral parent training: review of the literature and implications for practice. Clin Child Fam Psychol Rev. (2016) 19:203–15. 10.1007/s10567-016-0205-2 27311693

[13] Ofonedu ME Belcher HME Budhathoki C Gross DA. Understanding barriers to initial treatment engagement among underserved families seeking mental health services. J Child Fam Stud. (2017) 26:863–76. 10.1007/s10826-016-0603-6 28584498 PMC5456294

[14] Schneider BW Gerdes A Haack LM Lawton KE. Predicting treatment dropout in parent training interventions for families of school-aged children with ADHD. Child Fam Behav Ther. (2013) 35:144–69. 10.1080/07317107.2013.789365

[15] Swift JK Whipple JL Sandberg P. A prediction of initial appointment attendance and initial outcome expectations. Psychotherapy. (2012) 49(4):549–56. 10.1037/a0029441 22889099

[16] Dantas LF Fleck JL Cyrino Oliveira FL Hamacher S. No-shows in appointment scheduling—a systematic literature review. Health Policy. (2018) 122:412–21. 10.1016/j.healthpol.2018.02.002 29482948

[17] Sherman ML Barnum DD Buhman-Wiggs A Nyberg E. Clinical intake of child and adolescent consumers in a rural community mental health center: does wait-time predict attendance? Community Ment Health J. (2009) 45:78–84. 10.1007/s10597-008-9153-8 18807182

[18] Wang M-N Sandberg J Zavada Z Mittal M Gosling A Rosenberg T “Almost there” …why clients fail to engage in family therapy: an exploratory study. Contemp Fam Ther. (2006) 28:211–24. 10.1007/s10591-006-9001-3

[19] Dadds MR Sicouri G Piotrowska PJ Collins DAJ Hawes DJ Moul C Keeping parents involved: predicting attrition in a self-directed, online program for childhood conduct problems. J Clin Child Adolesc Psychol. (2019) 48(6):881–93. 10.1080/15374416.2018.1485109 30067388

[20] Chacko A Wymbs BT Rajwan E Wymbs F Feirsen N. Characteristics of parents of children with ADHD who never attend, drop out, and complete behavioral parent training. J Child Fam Stud. (2017) 26:950–60. 10.1007/s10826-016-0618-z

[21] Power TJ Hughes CL Helwig JR Nissley-Tsiopinis J Mautone JA Lavin HJ. Getting to first base: promoting engagement in family-school intervention for children with ADHD in urban, primary care practice. School Ment Health. (2010) 2:52–61. 10.1007/s12310-010-9029-2

[22] Stern SB Walsh M Mercado M Levene K Pepler DJ Carr A When they call, will they come? A contextually responsive approach for engaging multistressed families in an urban child mental health center: a randomized clinical trial. Res Soc Work. (2015) 25(5):549–63. 10.1177/1049731514548038

[23] Weaver A Greeno CG Goughler DH Yarzebinski K Zimmerman T Anderson C. The impact of system level factors on treatment timeliness: utilizing the Toyota production system to implement direct intake scheduling in a semi-rural community mental health clinic. J Behav Health Serv Res. (2013) 40(3):294–305. 10.1007/s11414-013-9331-5 23576137 PMC3732800

[24] Becker KD Wu EG Hukill A Brandt N Chorpita BF. How do mental health providers assess treatment engagement in youth and caregivers? J Child Fam Stud. (2021) 30:2527–38. 10.1007/s10826-021-02042-x

[25] Coatsworth JD Duncan LG Pantin H Szapocznik J. Patterns of retention in a preventive intervention with ethnic minority families. J Prim Prev. (2006) 27(2):171–93. 10.1007/s10935-005-0028-2 16532263 PMC1480362

[26] Quetsch LB Girard EI McNeil CB. The impact of incentives on treatment adherence and attrition: a randomized controlled trial of parent-child interaction therapy with a primarily latinx, low-income population. Child Youth Serv Rev. (2020) 112:104886. 10.1016/j.childyouth.2020.104886

[27] Fawley-King K Haine-Schlagel R Trask EV Zhang J Garland AF. Caregiver participation in community-based mental health services for children receiving outpatient care. J Behav Health Serv Res. (2013) 40(2):180–90. 10.1007/s11414-012-9311-1 23250770 PMC3625670

[28] Koerting J Smith E Knowles MM Latter S Elsey H McCann DC Barriers to, and facilitators of, parenting programmes for childhood behaviour problems: a qualitative synthesis of studies of parents’ and professionals’ perceptions. Eur Child Adolesc Psychiatry. (2013) 22:653–70. 10.1007/s00787-013-0401-2 23564207 PMC3826057

[29] Thomas R Abell B Webb HJ Avdagic E Zimmer-Gembeck MJ. Parent-child interaction therapy: a meta-analysis. Pediatrics. (2017) 140(3):e20170352. 10.1542/peds.2017-0352 28860132

[30] Kennedy SC Kim JS Tripodi SJ Brown SM Gowdy G. Does parent–child interaction therapy reduce future physical abuse? A meta-analysis. Res Soc Work Pract. (2016) 26(2):147–56. 10.1177/1049731514543024

[31] U.S. Census Bureau. Miami-Dade County, Florida. (2020). Available online at: https://data.census.gov/profile/Miami-Dade_County,_Florida?g=050XX00US12086#populations-and-people (Accessed August 9, 2025).

[32] Eyberg SM Funderburk BW. Parent-Child Interaction Therapy Protocol. Gainesville, FL: PCIT International (2011).

[33] Harris PA Taylor R Thielke R Payne J Gonzalez N Conde JG. Research electronic data capture (REDCap)—a metadata-driven methodology and workflow process for providing translational research informatics support. J Biomed Inform. (2009) 42:377–81. 10.1016/j.jbi.2008.08.010 18929686 PMC2700030

[34] Harris R Taylor BL Minor V Elliott M Fernandez L O’Neal L The REDCap consortium: building an international community of software partners. J Biomed Inform. (2019) 95. 10.1016/j.jbi.2019.103208 31078660 PMC7254481

[35] Eyberg SM Pincus D. Eyberg Child Behavior Inventory and Sutter-Eyberg Student Behavior Inventory–Revised: Professional Manual. Odessa, FL: Psychological Assessment Resources (1999).

[36] Gross D Fogg L Young M Ridge A Cowell J Sivan A Reliability and validity of the Eyberg child behavior inventory with African-American and Latino parents of young children. Res Nurs Health. (2007) 30(2):213–23. 10.1002/nur.20181 17380522

[37] Reynolds CR Kamphaus RW. Behavior Assessment System for Children Manual. 3rd ed. San Antonio, TX: Pearson (2015).

[38] Hosmer DW Lemeshow S Sturdivant RX. Applied Logistic Regression. 3rd ed. Hoboken, NJ: Wiley (2013).

[39] Peduzzi P Concato J Kemper E Holford TR Feinstein AR. A simulation study of the number of events per variable in logistic regression analysis. J Clin Epidemiol. (1996) 49(12):1373–9. 10.1016/S0895-4356(96)00236-3 8970487

[40] Andrade LH Alonso J Mneimneh Z Wells JE Al-Hamzawi A Borges G Barriers to mental health treatment: results from the WHO world mental health surveys. Psychol Med. (2014) 44:1303–17. 10.1017/S0033291713001943 23931656 PMC4100460

[41] Fernandez MA Butler AM Eyberg SM. Treatment outcome for low socioeconomic status African American families in parent-child interaction therapy: a pilot study. Child Fam Behav Ther. (2011) 33(1):32–48. 10.1080/07317107.2011.545011

[42] Chavez FT Stapert ECR Fernandez M. DPICS in color: rethinking the English language and illuminating the PRIDE in the richness of urban vernacular. In: 2021 PCIT International Biennial Convention, May 25–27, 2021. Orlando, FL (2021).

[43] Jent J Rothenberg WA Peskin A Acosta J Weinstein A Concepcion R Clinical approaches, treatment formats, and predictors of success for an 18-week model of parent-child interaction therapy. Front Psychol. (2024) 14. 10.3389/fpsyg.2023.1233683 PMC10616824 37915519

[44] McCabe KM Yeh M. Parent-child interaction therapy for Mexican Americans: a randomized clinical trial. J Clin Child Adolesc Psychol. (2009) 38(5):753–9. 10.1080/15374410903103544 20183659

[45] Green Rosas Y McCabe KM Zerr A Yeh M Gese K Barnett ML. Examining English-and Spanish-speaking therapist behaviors in parent–child interaction therapy. Int J Environ Res Public Health. (2022) 19:1–12. 10.3390/ijerph19084474 PMC9031310 35457342

[46] Yeh M McCabe K Hurlburt M Hough R Hazen A Culver S Referral sources, diagnoses, and service types of youth in public outpatient mental health care: a focus on ethnic minorities. J Behav Health Serv Res. (2002) 29(1):45–60. 10.1007/BF02287831 11840904

[47] McCabe KM Yeh M Garland AF Lau AS Chavez G. The GANA program: a tailoring approach to adapting parent-child interaction therapy for Mexican Americans. Educ Treat Children. (2005) 28(2):111–29.

[48] Abrahamse ME Niec LN Junger M Boer F Lindauer RJL. Risk factors for attrition from an evidence-based parenting program: findings from The Netherlands. Child Youth Serv Rev. (2016) 64:42–50. 10.1016/j.childyouth.2016.02.025

[49] Fernandez MA Eyberg SM. Predicting treatment and follow-up attrition in parent-child interaction therapy. J Abnorm Child Psychol. (2009) 37(3):431–41. 10.1007/s10802-008-9281-1 19096926

[50] Shadish WR Cook TD Campbell DT. Experimental and Quasi-Experimental Designs for Generalized Causal Inference. Houghton: Mifflin and Company (2002).

